# Potent Antifungal Activity of Penta-*O*-galloyl-β-d-Glucose against Drug-Resistant *Candida albicans*, *Candida auris*, and Other Non-*albicans Candida* Species

**DOI:** 10.1021/acsinfecdis.3c00113

**Published:** 2023-08-22

**Authors:** Lewis Marquez, Yunjin Lee, Dustin Duncan, Luke Whitesell, Leah E. Cowen, Cassandra Quave

**Affiliations:** † Molecular and Systems Pharmacology, Laney Graduate School, Emory University, Atlanta, Georgia 30322, United States; ‡ Jones Center at Ichauway, Newton, Georgia 39870, United States; § Department of Molecular Genetics, University of Toronto, Toronto, Ontario M5G 1M1, Canada; ∥ Department of Chemistry, Brock University, St. Catharines, Ontario L2S 3A1, Canada; ⊥ Center for the Study of Human Health, Emory University, Atlanta, Georgia 30322, United States; # Department of Dermatology, Emory University, Atlanta, Georgia 30322, United States

**Keywords:** natural product, candidiasis, mechanism of action, iron chelation

## Abstract

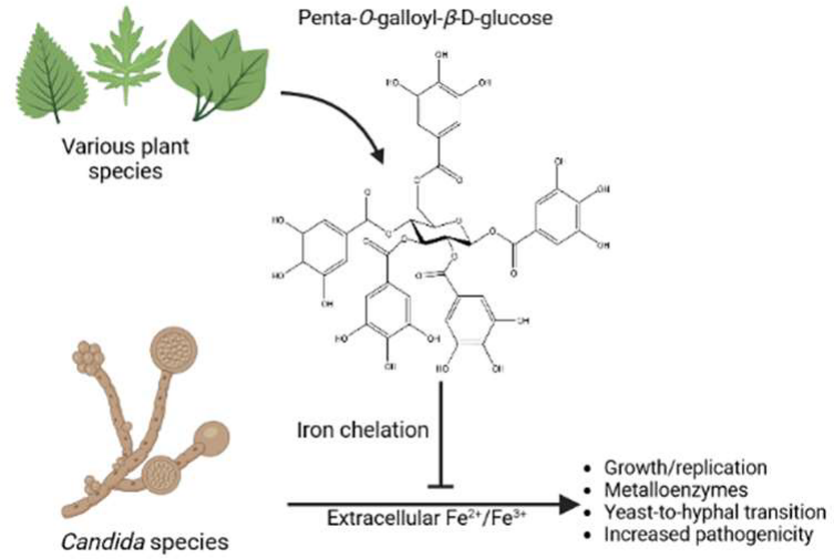

Among fungal pathogens, infections by drug-resistant *Candida* species continue to pose a major challenge to healthcare. This study aimed to evaluate the activity of the bioactive natural product, penta-*O*-galloyl-β-d-glucose (PGG) against multidrug-resistant (MDR) *Candida albicans*, MDR *Candida auris*, and other MDR non-*albicans Candida* species. Here, we show that PGG has a minimum inhibitory concentration (MIC) of 0.25–8 μg mL^–1^ (0.265–8.5 μM) against three clinical strains of *C. auris* and a MIC of 0.25–4 μg mL^–1^ (0.265–4.25 μM) against a panel of other MDR *Candida* species. Our cytotoxicity studies found that PGG was well tolerated by human kidney, liver, and epithelial cells with an IC_50_ > 256 μg mL^–1^ (>272 μM). We also show that PGG is a high-capacity iron chelator and that deletion of key iron homeostasis genes in *C. albicans* rendered strains hypersensitive to PGG. In conclusion, PGG displayed potent anti-*Candida* activity with minimal cytotoxicity for human cells. We also found that the antifungal activity of PGG is mediated through an iron-chelating mechanism, suggesting that the compound could prove useful as a topical treatment for superficial *Candida* infections.

*Candida* is a genus of commensal yeast that can most often be found in the digestive tract and colonizing the skin of healthy individuals.^1,2^ Commensal *Candida* species can lead to superficial skin candidiasis, with prevalence varying by the geographic region, with reported rates ranging from 22% (China), 40% (Iran), and up to 56% (Serbia).^3−5^ When this yeast develops resistance to antifungals and/or invades other parts of the human body, termed invasive candidiasis, serious issues can arise. Invasive candidiasis is often detected as candidemia, a bloodstream infection, but can also involve other major organs such as the heart, kidneys, spleen, and liver.^6^ There are an estimated 25,000 infections attributed to *Candida* each year in the USA.^7^ Studies in the USA and Spain have estimated a 29–31% mortality rate for invasive candidiasis.^8,9^ Other studies found mortality rates to be twice as high in South Africa (60%) and Brazil (72%).^10,11^

*Candida auris*, first identified in 2009, is a relatively new *Candida* species emerging in the landscape of invasive candidiasis. *C. auris* infections are highly invasive and deadly,^12,13^ with mortality rates as high as 60%.^14,15^ Most recently, from June 2020 to May 2021, there were more than 1,000 clinical cases of *C. auris* reported across 19 states in the USA.^16^

Drug resistance and a paucity of new antifungals in development necessitate the discovery of new anti-*Candida* drugs. Preparations from *Schinus terebinthifolia* Raddi (Anacardiaceae family), the Brazilian peppertree, have traditionally been used as an antiseptic and for the treatment of skin ulcers and wound healing.^17^ The present study investigated a bioactive small molecule with anti-*Candida* activity that our lab first isolated from the leaves of *S. terebinthifolia*—the ubiquitous natural product penta-*O*-galloyl-β-d-glucose (PGG).^18^ PGG is a hydrolyzable tannin with a plethora of biological activities such as antibacterial, anticancer, and antiviral activities (Table 1). We examined PGG for cytotoxicity against a panel of human cell lines and growth inhibition against multidrug-resistant (MDR) strains of *Candida albicans*, *Candida glabrata*, *Candida parapsilosis*, and *C. auris* (Table 2). Additionally, we sought to elucidate the mechanism of action for the antifungal activity of PGG.

**Table 1:** Notable Biological Activities of Penta-*O*-galloyl-β-d-glucose (PGG)

target	IC_50_/EC_50_	mode of action	refs
**Antibacterial**			
*Staphylococcus aureus* (various strains)	16–32 μg/mL	possibly iron chelation	(19)
MRSA (various strains)	16–32 μg/mL	possibly iron chelation	(19)
*Acinetobacter baumannii* (various strains)	16–64 μg/mL	possibly iron chelation	(18)
**Anticancer (Liver Cancer)**			
SK-HEP-1 cells	25–50 μM	inhibition of proliferation (inactivation of NF-κB)	(20)
**Anticancer (Colorectal Cancer)**			
HCT-116 cells	1.61 μM	inhibition of proliferation (induction of the *p*53 gene)	(21, 22)
HT-29 cells	4.46 μM	inhibition of proliferation (induction of the *p*53 gene)	(21, 22)
**Anticancer (Breast Cancer)**			
MDA-MB-231	50.23 μM	induction of apoptosis	(23)
MDA-MB-468	35.72 μM	induction of apoptosis	(23)
**Antiviral**			
influenza A	5.3 μg/mL	interference with viral replication	(24)

**Table 2:** MIC Values for PGG against the Various *Candida* Species and Strains

			IC_50_ (μg/mL)	MIC (μg/mL)	MIC (μg/mL)		
species	strain ID	antibiogram	PGG	PGG	AMB	FLC	KTC
*Candida albicans*	ATCC 90028	Afg^S^, Cas^S^, Flc^S^, Itc^S^, Mfg^S^, Vrc^S^	0.5	1	1	0.25	<0.0039
*Candida albicans*	MH12	Afg^S^, Cas^S^, Flc^R^, Itc^S^, Mfg^S^, Vrc^S^	0.25	0.5	1	>8	1
*Candida parapsilosis*	AR Bank #0335	Afg^I^, Cas^S^, Flc^R^, Mfg^S^, Vrc^R^	1	4	1	>8	4
*Candida parapsilosis*	AR Bank #0337	Afg^S^, Cas^S^, Flc^R^, Mfg^S^, Vrc^R^	0.5	0.5	1	>8	2
*Candida parapsilosis*	AR Bank #0338	Afg^S^, Cas^S^, Flc^R^, Mfg^S^	0.25	0.5	1	>8	0.5
*Candida parapsilosis*	AR Bank #0339	Afg^S^, Cas^S^, Flc^R^, Mfg^S^	0.5	1	2	>8	0.25
*Candida glabrata*	AR Bank #0325	Afg^R^, Cas^R^, Flc^R^, Mfg^R^	0.25	0.5	1	>8	4
*Candida glabrata*	AR Bank #0326	Afg^S^, Cas^S^, Flc^SDD^, Mfg^S^,	0.125	0.25	1	4	0.25
*Candida auris*^a^	AR Bank #0381	Afg^S^, Amb^S^, Cas^S^, Flc^S^, Mfg^S^	—	0.25	1	4	<0.0039
*Candida auris*^a^	AR Bank #0387	Afg^S^, Amb^S^, Cas^S^, Flc^I^, Mfg^S^	0.5	1	0.5	1	<0.0039
*Candida auris*^a^	AR Bank #0390	Afg^S^, Amb^R^, Cas^S^, Flc^R^, Mfg^S^	4	8	2	>8	0.5

a There are currently no established *C. auris* antifungal breakpoints; as outlined by the CDC,^25^ breakpoints from closely related *Candida* sp. were used to determine the antibiogram profile. Abbreviations used are amphotericin B (Amb), anidulafungin (Afg), caspofungin (Cas), itraconazole (Itc), fluconazole (Flu), micafungin (Mfg), voriconazole (Vrc), and ketoconazole (Ktc). Superscripts used: S, susceptible; SDD, susceptible dose-dependent; I, intermediate; and R, resistant.

## Results

### Anti-*Candida* Activity of Penta-*O*-galloyl-β-d-glucose

**Figure 1:**
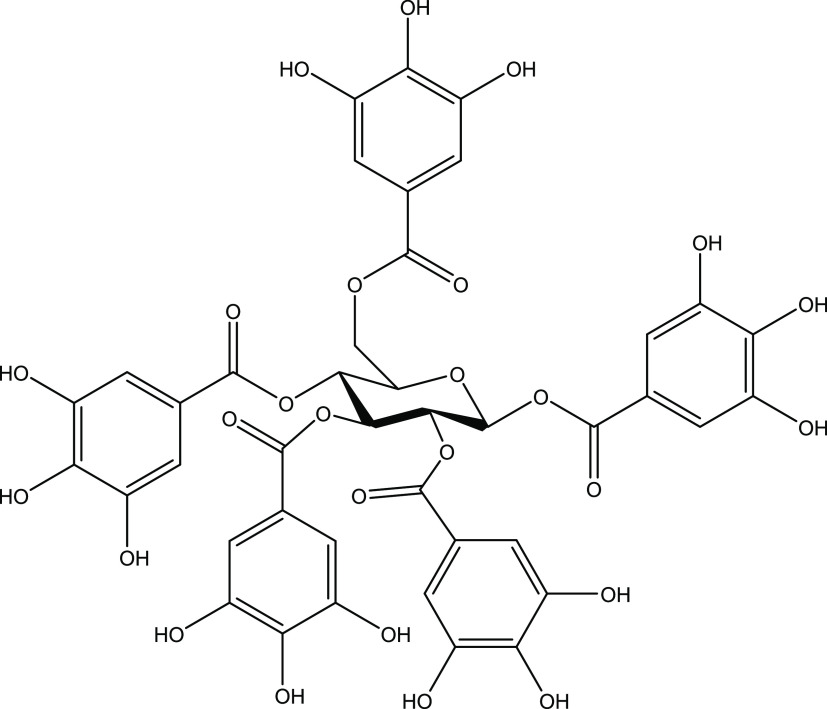
Chemical structure of penta-*O*-galloyl-β-d-glucose, PGG.

**Figure 2:**
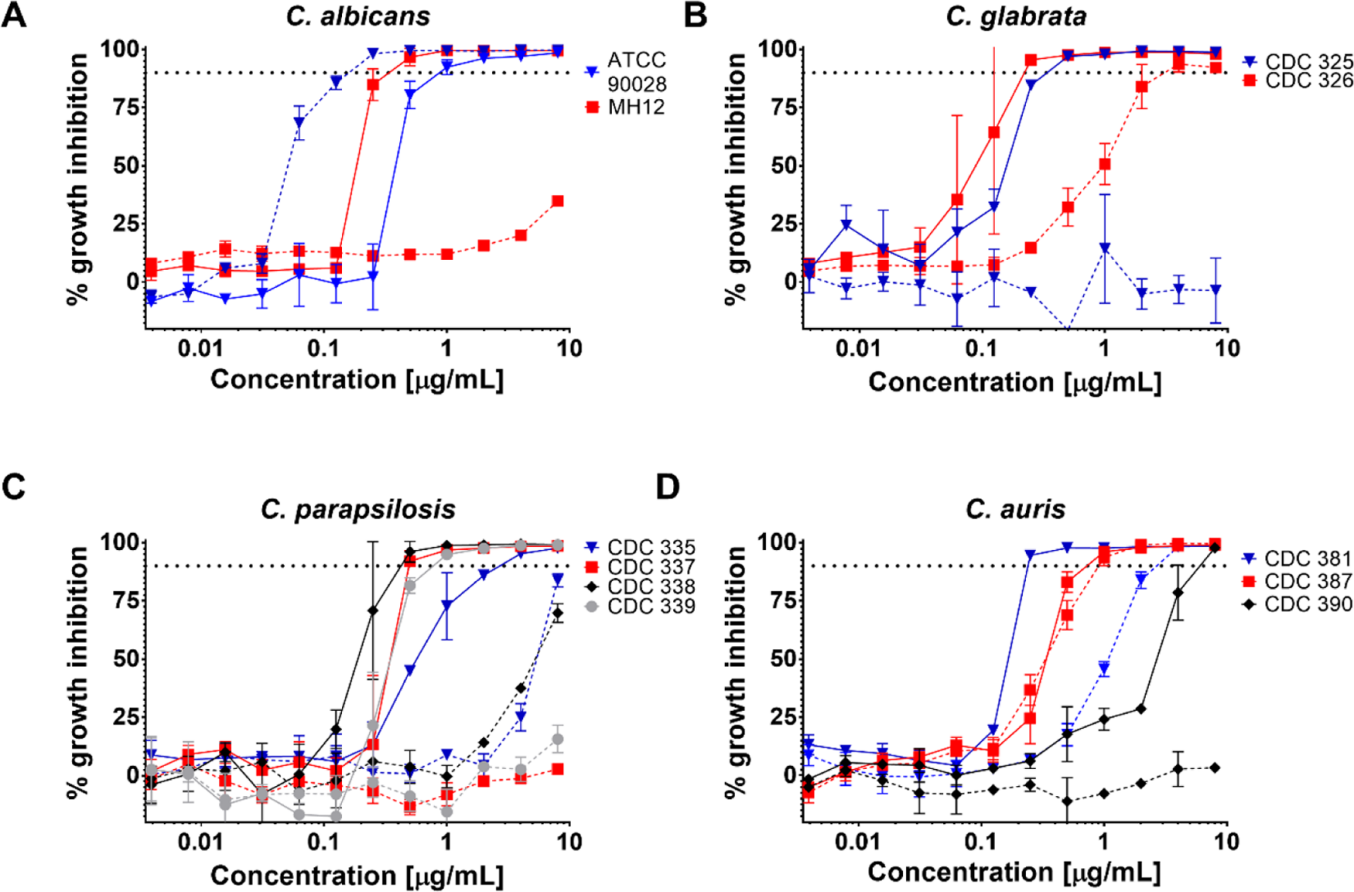
Penta-*O*-galloyl-β-d-glucose displays broad spectrum anti-*Candida* activity. Growth inhibition of PGG against (A) *C. albicans*, (B) *C. glabrata*, (C) *C. parapsilosis*, and (D) *C. auris*. Solid lines represent PGG. Dashed lines represent the positive control fluconazole. The data are plotted as the mean % growth inhibition ± SD as compared to the DMSO vehicle from two independent experiments. The horizontal dotted line represents 90% growth inhibition. See Supplemental Figures S8–S11 for expanded graphs including additional positive controls. See Table 2 for strain details.

Penta-*O*-galloyl-β-d-glucose (PGG, Figure 1) was tested for growth inhibition using Clinical and Laboratory Standards Institute (CLSI) guidelines against seven drug-resistant strains and four susceptible strains from four species of *Candida*: two strains of *C. albicans*, two strains of *C. glabrata*, four strains of *C. parapsilosis*, and three strains of *C. auris* (Table 2). We found that MICs against all *Candida* species tested ranged from 0.25–8 μg mL^–1^ (equivalent to 0.265–8.5 μM) (Table 2). PGG MICs for *C. albicans* ranged from 0.5 to 1.0 μg mL^–1^ (Figure 2A), for *C. glabrata* ranged from 0.25 to 0.5 μg mL^–1^ (Figure 2B), *for C. parapsilosis* ranged from 0.5 to 4.0 μg mL^–1^ (Figure 2C), and for *C. auris* ranged from 0.25 to 8.0 μg mL^–1^ (Figure 2D).

We also examined PGG for the synergistic interaction with two common antifungals and found that PGG does not potentiate the activity of the echinocandin caspofungin or the azole fluconazole against *C. albicans* (Supplemental Figure S1).

### Cytotoxicity

We examined PGG’s effects on cell viability and cytotoxicity against three human cell lines: human embryonic kidney 293 cells (Hek293), human hepatoblastoma cells (HepG2), and human epidermal keratinocytes (HaCaT). In cytotoxicity studies with Hek293, HepG2, and HaCaT cells, we found that PGG was well tolerated with a CC_50_ > 256 μg mL^–1^ (>272 μM) (Supplemental Figure S2). We assessed relative viable cell number by measuring relative ATP levels *in vitro* after treatment with PGG and found that PGG reduced the viable cell number by 50% in Hek293 and HepG2 cells at an IC_50_ of 64 μg mL^–1^ (68 μM) and displayed an IC_50_ of 32 μg mL^–1^ (34 μM) against HaCaT cells (Supplemental Figure S3).

### PGG Sensitivity of *C. albicans* Is Not Modulated by Major Efflux Pumps

**Figure 3:**
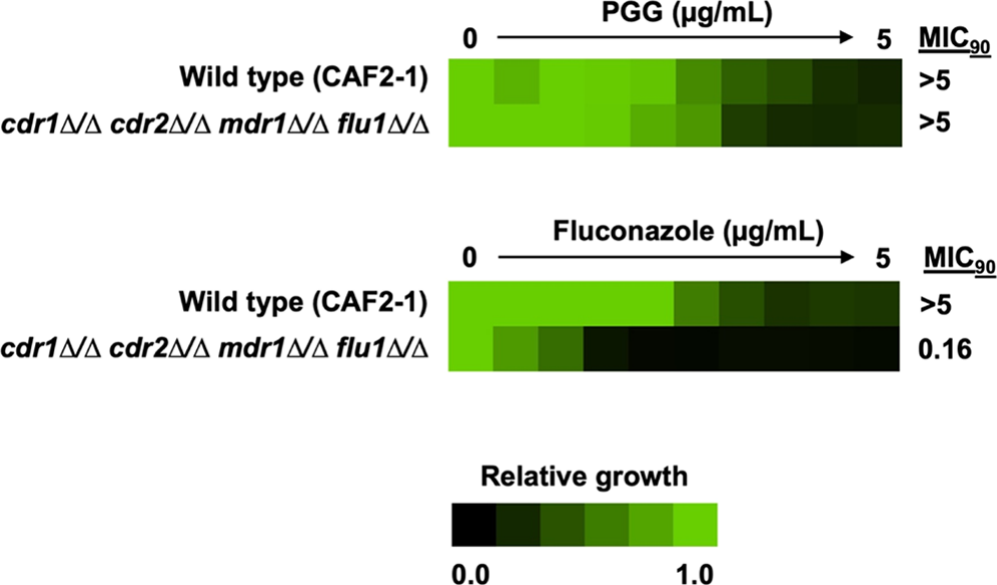
PGG sensitivity in *C. albicans* is not modulated by efflux pumps. The *cdr*1Δ/Δ, *cdr*2Δ/Δ, *mdr*1Δ/Δ, *flu*1Δ/Δ, and wild-type strains were grown overnight in YPD and subject to dose–response assays in RPMI-1640 medium with twofold dilution gradients of PGG. Fluconazole was included as a positive control. Growth (OD_600nm_) was measured after 72 h of incubation at 30 °C, averaged between technical duplicates, and normalized to the drug-free wild-type control. Results are presented in a heat-map format with a scale bar provided at the bottom of the figure. All assays were performed in biological duplicates.

To determine if PGG activity was affected by efflux mediated by four of the major *C. albicans* efflux pumps, we tested PGG against a mutant strain of *C. albicans* with deletion of the genes encoding these efflux pumps: *Candida* drug-resistance genes (*CDR*1, *CDR*2), fluconazole resistance gene (*FLU*1), and (*MDR*1) multidrug resistance gene.^26^ We found that the absence of these major efflux pumps did not modify sensitivity to PGG, in contrast to the hypersensitivity seen with fluconazole, a compound known to undergo efflux pump-mediated transport (Figure 3). These data provide evidence that PGG is not a substrate for these major *C. albicans* efflux pumps and/or does not need to enter the cell to produce its effect.

### PGG Does Not Bind to Fungal Ergosterol

Probing for possible mechanisms of action, we examined the interaction of PGG with ergosterol, a sterol found in the fungal cell membrane and the major target of the polyene class of antifungals. We found that PGG’s growth inhibitory activity was not affected by additional ergosterol, providing evidence that PGG does not target ergosterol in the fungal cell membrane (Supplemental Figure S4).

### PGG’s Anti-Candida Activity Is Tied to Iron Chelation

**Figure 4:**
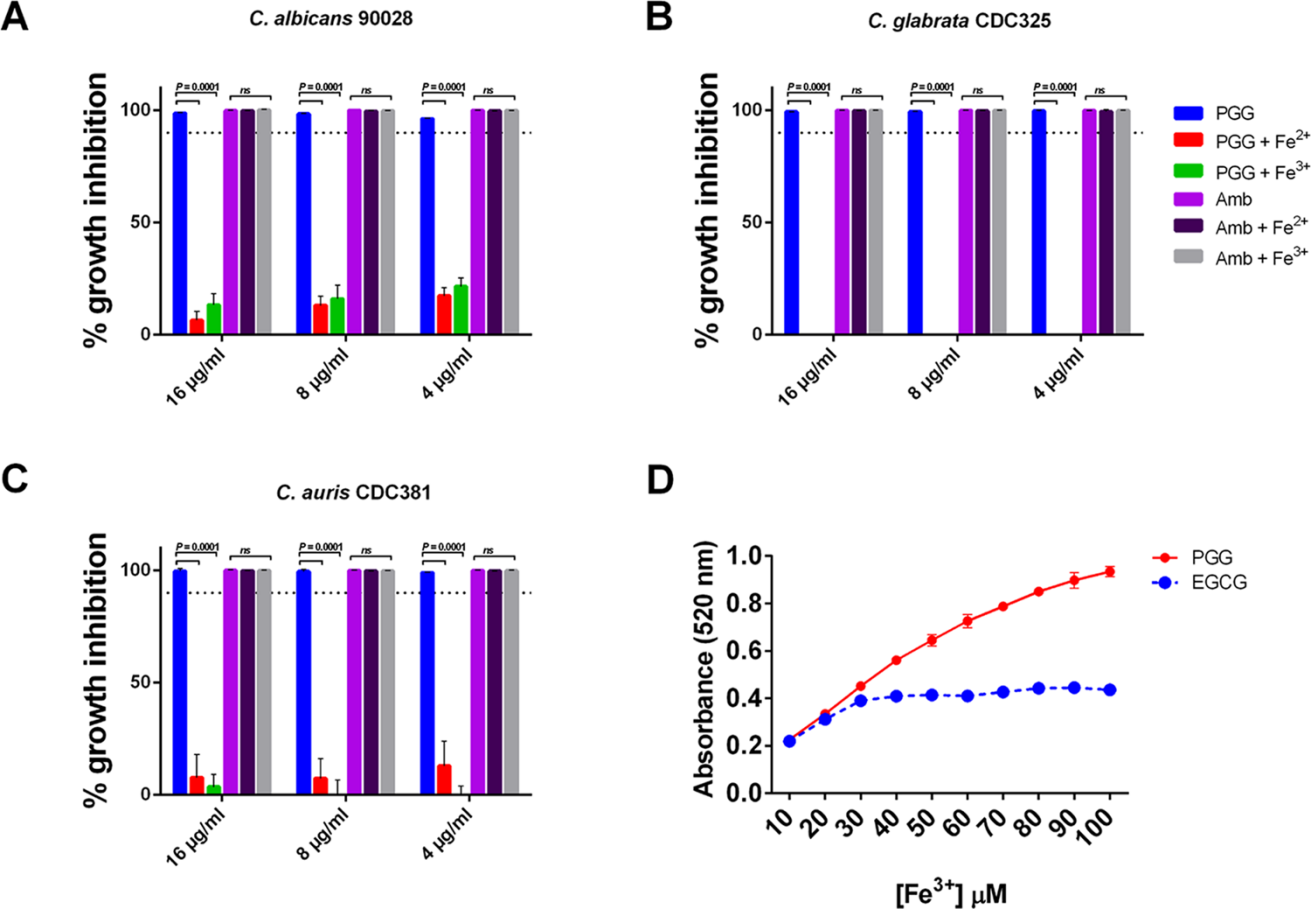
Growth inhibition of PGG against multiple *Candida* species is abrogated by iron supplementation, and PGG binds multiple iron ions. (A) *C. albicans* 90028, (B) *C. glabrata* CDC325, and (C) *C. auris* CDC381 were treated with PGG or Amphotericin B at varying concentrations (4, 8, and 16 μg/mL) and media was supplemented with a 10 mM solution of ferrous sulfate (Fe^2+^) or ferric sulfate (Fe^3+^) and % growth inhibition measured after 48 h. The data are plotted as the mean % growth inhibition ± SD as compared to the DMSO vehicle from two independent experiments. The horizontal dotted line represents 90% growth inhibition compared to the vehicle. Statistical analysis was done using two-way ANOVA with multiple comparisons. (D) PGG or EGCG was allowed to react with varying concentrations of iron (III) in solution at pH 2.0. The bipy solution was added to the mixtures and the formation of the [Fe(bipy)_3_]^2+^ complex was measured at 520 nm. [PGG]_total_, [EGCG]_total_ = 10 μM. The data are plotted as the mean absorbance ± SD from two independent experiments.

A previous study by Cho et al. (2010) found a possible link between the antibacterial activity of PGG and iron chelation in cultures of *Staphylococcus aureus*.^19^ To determine if iron chelation was also relevant to the antifungal activity of PGG against *Candida*, we tested *C. albicans*, *C. glabrata*, and *C. auris* grown in iron-supplemented media. We found that the inhibitory effect of PGG against *C. albicans*, *C. glabrata*, and *C. auris* was abrogated upon the addition of iron supplementation (Figure 4A–C). *C. albicans*, *C. glabrata*, and *C. auris* supplemented with 10 mM of iron (II) or iron (III) led to a reduction of approximately 80–100% of the growth inhibitory activity of PGG at concentrations of 4–16 μg mL^–1^. We next examined the specificity of PGG for other metals and cations such as manganese, zinc, calcium, and magnesium. We found no significant effect on growth inhibition from cation treatment, no effect from zinc supplementation, and a minor effect on growth inhibition from manganese supplementation at 0.5× MIC of PGG (Supplemental Figures S5, S6). These results provide evidence that PGG’s activity is selective for iron.

To further investigate the role of iron chelation as it relates to PGG’s anti-*Candida* activity, we next examined the iron-binding capacity of PGG. We utilized the ligand 2,2′-bipyridyl (bipy). The bipy method utilized a proxy of PGG-iron binding through the spectroscopic determination of bipy–iron complex formation. Bipy is a well-known ligand that can measure, from mixtures of iron (III) and a reducing agent, the amount of free iron (II) as a red-colored complex [Fe(bipy)_3_]^2+^ with absorbance at 520 nm. From this experiment, we can calculate the amount of iron (III) ions bound by the galloyl moieties on the PGG molecule through the reduction of iron (III) to iron (II) and the detection of the colored [Fe(bipy)_3_]^2+^ complex in a 1:1 ratio. Results of the 2,2′-bipyridyl test found that in the presence of excess iron (III), PGG bound more than 2× the amount of iron (III) compared to epigallocatechin-gallate (EGCG), as shown in Figure 4D. EGCG is a natural product with two galloyl side groups, as compared to the five galloyl side groups on PGG. EGCG has previously been shown to bind up to two iron (III) ions per molecule of EGCG.^27^ Calculations of these results equate to the reduction of 10 Fe^3+^ ions to 10 Fe^2+^ ions and the binding of 5 iron (III) ions per PGG molecule at the highest tested concentrations. We also attempted to determine stoichiometry using Job’s method.^28,29^ Interestingly, we found that depending on the mole fraction of iron and PGG, the peak wavelength varied (Supplemental Figure S7A). This suggests that at different mole fractions of iron and PGG, different complexes form. Since Job’s method assumes that only one complex is formed, we were unable to determine a discrete stoichiometry of PGG to iron but were still able to demonstrate that PGG was able to bind multiple iron ions (Supplemental Figure S7B). These results provide evidence for the high iron-binding capacity of each PGG molecule and the ability to form multiple complexes with iron.

**Figure 5:**
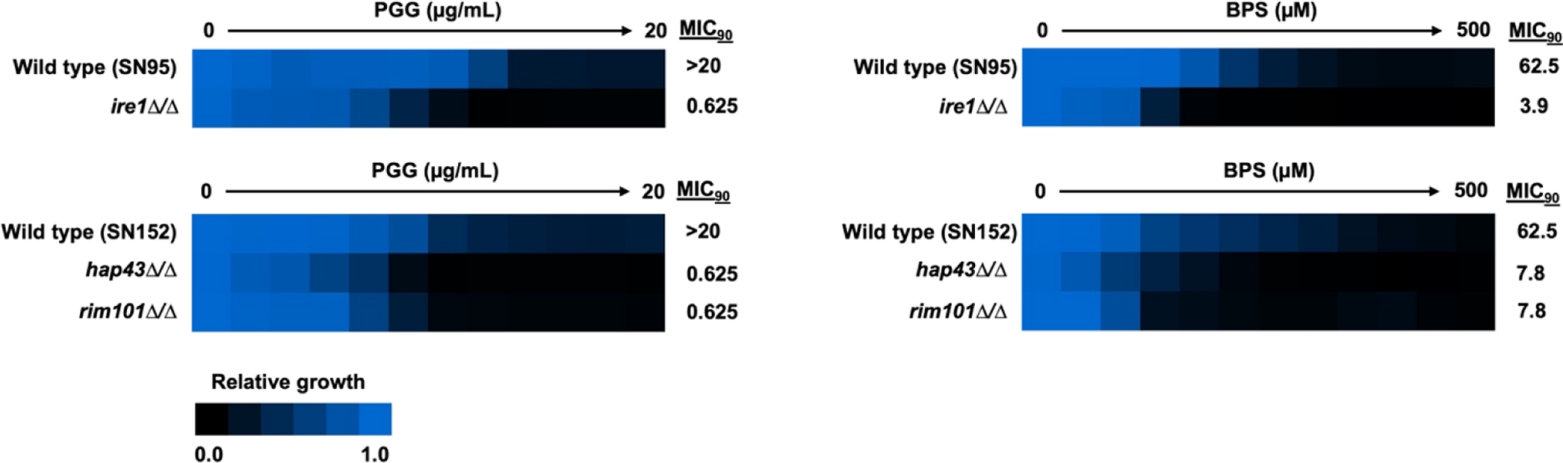
Iron homeostasis genes are required for PGG tolerance in *C. albicans*. The *ire*1Δ/*ire*1Δ, *hap*43Δ/*hap*43Δ, and *rim*101Δ/*rim*101Δ strains and their respective wild-type controls were grown overnight in YPD and subject to dose–response assays in RPMI-1640 medium with twofold dilution gradients of PGG. The metal chelator BPS was included as a positive control. After 48 h incubation at 30 °C, the relative viable cell number was assessed. Data were averaged between technical duplicates and normalized to wild-type drug-free control wells. Results are presented in a heat-map format with a color scale bar presented at the bottom of the figure. All assays were performed in biological duplicates.

Given that iron rescues PGG growth inhibition, we next examined whether key iron homeostasis genes in *C. albicans* are required for basal tolerance to PGG. We tested PGG’s effect against multiple iron homeostasis gene mutants and found that deletion of *IRE*1, which encodes a protein kinase with an essential function in iron uptake, as well as *HAP*43 and *RIM*101, which encode transcription factors involved in the iron-starvation response,^30−32^ rendered strains hypersensitive to PGG, as is the case with a known iron chelator, bathophenanthrolinedisulfonate (BPS) (Figure 5). Together, these results support the conclusion that PGG’s antifungal activity is linked to iron chelation.

## Discussion

Penta-*O*-galloyl-β-d-glucose (PGG) is a hydrolyzable tannin with many functional roles, such as antibacterial, anticancer, and antiviral activities (Table 1). This study is the first to investigate PGG’s antifungal activity against a panel of multiple *Candida* species. Our findings show that PGG displays potent activity against *C. auris*, *C. albicans*, *C. glabrata*, and *C. parapsilosis* with MICs in the range of 0.25–8 μg mL^–1^ (0.265–8.5 μM). Our findings show that PGG is comparable to, or more effective than, conventional antifungals against multiple drug-resistant strains of *Candida* species when tested under standard microbiological assay conditions.

We found that PGG displayed a low level of *in vitro* cytotoxicity (CC_50_ > 256 μg mL^–1^) against human kidney, liver, and skin cells in a serum-supplemented tissue culture medium. For a majority of *Candida* species and strains tested, PGG displayed a MIC between 0.25–1 μg mL^–1^ (0.265–1.06 μM). However, after further testing of PGG against drug-sensitive *C. albicans* in fungal growth media supplemented with 10% fetal bovine serum (FBS), we saw evidence of serum-induced MIC reversal and the loss of growth inhibition of PGG at concentrations up to 512 μg mL^–1^ (data not shown). Of note, the presence of FBS in our human cell cytotoxicity studies could have influenced the results of our cytotoxicity assays through iron supplementation or protein binding of PGG, potentially masking any cytotoxic effects. Despite these concerns, while we did not investigate the *in vivo* tolerability of PGG, another study found PGG to be tolerated without ill effects at doses of 20 mg/kg/day orally in mice, and a separate study found a dose up to 120 mg/kg/day intravenously in rats produced no ill effects.^33,34^ Most recently, PGG was used in humans to safely treat abdominal aortic aneurysms via a balloon catheter at a rate of 3 mg/mL over 25 mL.^35^ Our findings, coupled with the aforementioned *in vivo* studies, suggest that PGG is a compound that is well tolerated both *in vitro* and *in vivo*.

One major weakness of PGG is the high clearance rate and breakdown of the compound *in vivo*. A separate study found that PGG exhibited a high efflux ratio, which provides evidence that PGG likely undergoes active transport out of the cell.^36^ Another study found that PGG could not be detected in the plasma after oral administration in mice.^37^ The breakdown of PGG into various gallic acid derivatives and metabolites *in vivo* may be the reason PGG was below the level of detection in plasma and may contribute to its high clearance rate *in vivo*.^38^ For these reasons, PGG, in its current form, likely makes a poor candidate as a drug for systemic administration. However, in line with the traditional ethnobotanical topical use of the leaves of *S. terebinthifolia*, the broad anti-*Candida* activity of PGG merits further examination *in vivo* to evaluate any potential as a topical antifungal agent.

The mechanism of action for the antimicrobial effect of PGG is likely attributable to its activity as an iron chelator. We expect that iron binding occurs via the hydroxyl groups on the galloyl moieties, a trait shared with epigallocatechin-gallate^27^ and other similarly structured compounds.^39^ Iron is essential for most living organisms, as it is a key component in a variety of metalloenzymes for processes such as cell growth, electron transport, and DNA synthesis. Iron is also essential for the growth and survival of many fungi, including *Candida*.^40−42^ Uptake of extracellular iron, through whatever means, is a crucial process necessary for the growth and survival of *Candida* species. We observed that excess free iron rescues PGG growth inhibition and genetic deletion of key iron homeostasis genes such as *IRE*1, *HAP*43, and *RIM*101 in *C. albicans* conferred hypersensitivity to PGG. Our iron-binding experiment found that PGG strongly interacts with free iron, with each molecule of PGG binding up to five iron (III) ions. PGG’s iron-chelating ability is expected to be the mechanism through which PGG elicits its growth inhibitory effect against the tested *Candida* species. Preventing *Candida* species from acquiring this crucial micronutrient is an effective means of preventing their proliferation.

There is one other example of an iron-chelating antifungal, the 50-year-old topical antifungal drug ciclopirox.^43,44^ Despite the extensive use of ciclopirox as a topical antifungal for over 50 years, there have been no reported ciclopirox-resistant strains in the literature. While the mechanism of action for ciclopirox was initially unknown, there has been ample evidence pointing to its antifungal activity being mediated through an iron-chelating mechanism.^45−47^ Sigle et al.^47^ found that the antifungal activity of ciclopirox was abrogated upon the addition of both Fe^2+^ and Fe^3+^, similar to what we found to occur for our compound PGG. Importantly, PGG is superior to ciclopirox in its iron-binding capacity—three molecules of ciclopirox bind one iron ion, whereas we found one molecule of PGG binds up to five.^48^ Sigle et al.^47^ found multiple iron metabolism genes upregulated in response to ciclopirox treatment. One of these genes was the unknown transcription factor *IPF*7711, now known as *HAP*43.^49^*HAP*43 (alias *IPF*7711) is involved in the iron-starvation response^31^ and is one of the genes for which we found that deletion confers hypersensitivity to PGG in *C. albicans*. Regarding the development of resistance to an iron-chelating antifungal agent, Niewerth et al.^45^ found that ciclopirox resistance did not develop in *C. albicans* over 6 months of treatment *in vitro*. This is similar to the trend we found previously, in which the gram-negative bacteria *Acinetobacter baumanii* did not develop resistance to PGG over 21 daily passages.^18^ The continued clinical use of the iron-chelating topical antifungal ciclopirox, without the emergence of resistant strains, provides logical evidence that the iron-chelating compound PGG could also be developed into an effective topical antifungal agent.

As a final note, our review of the literature on the other biological activities of PGG (Table 1) leads us to suggest that iron chelation may also play a role in PGG’s anticancer activity. The authors Oh et al.^20^ found that PGG inhibited the activation of nuclear factor kappa B (NF-κB), a transcription factor involved in the regulation of inflammation and cell proliferation.^50^ We theorize that the inactivation of NF-κB by PGG may be due to the iron-chelating activity of PGG. It has been reported that intracellular iron is involved in the activation of NF-κB,^51^ and a separate study found that iron activates NF-κB in Kupffer cells.^52^ The downstream inactivation of NF-κB and the evident inhibition of proliferation in SK-HEP-1 hepatoma cells may be due to the chelating activity of PGG sequestering iron and modulation of upstream regulators of NF-κB. Other studies examining colorectal cancer cells found that PGG inhibits the proliferation of HCT-116 and HT-29 cells.^21^ PGG’s anticancer effect against colorectal cancer cells was proposed to be acting through induction of the tumor suppressor protein *p*53.^22^ Iron metabolism is reported to regulate the activation of *p*53. Studies have reported that iron depletion by iron chelators promotes activation of *p*53.^53,54^ It is possible, for some cancer types, that the anticancer activity of PGG may initially stem from iron chelation and the anticancer activity reported may be a downstream effect of iron depletion. PGG’s iron-chelating ability may be the basis for many of the biological roles reported for PGG, but more studies are needed to support this connection.

PGG’s strong anti-*Candida* activity and its demonstrated iron-chelating activity warrant further investigation as a topical therapeutic agent for use against *Candida* infections. A topical PGG formulation could be beneficial in the treatment of other *Candida* skin infections or fungal infections found in warm, moist creased skin environments such as the armpits and groin. Studies have shown relatively high recovery yields of PGG (36%) from *Mangifera indica* L. kernels and mango seeds, an unused food element that could greatly reduce acquisition costs.^55^ For these reasons, PGG’s properties as an anti-*Candida* agent warrant a deeper investigation into its potential therapeutic applications.

## Methods

### Cell Lines and Growth Conditions

Fungal strains tested were purchased from the American Type Culture Collection (ATCC) or acquired from the FDA-CDC Antimicrobial Resistance Isolate Bank. A full list of tested strains and their antimicrobial susceptibility profiles can be found in Table 2. Strains were maintained on BBL Sabouraud dextrose agar (SDA, BD) at 35 °C in a humidified chamber for 48 h before use. The *C. albicans* DSY1024 (*cdr*1Δ*/cdr*1Δ *cdr*2Δ*/cdr*2Δ *mdr*1Δ*/mdr*1Δ *flu*1Δ*/flu*1Δ) mutant,^26^*C. albicansire*1Δ/Δ mutant,^56^ and *C. albicanshap*43Δ/Δ *andC. albicansrim*101Δ/Δ mutants^57^ were obtained from previous studies. For all other assays, strains are listed in Supplemental Table S1, along with their sources. Strains were cultured in YPD (1% yeast extract, 2% peptone, and 2% glucose) at 30 °C.

Hek293 cells were purchased from BEI Resources. HaCaT cells were purchased from ATCC. HepG2 cells were provided by Dr. Edward Morgan (Emory University). Mammalian cell cultures were maintained in Dulbecco’s modified Eagle’s medium with L-glutamine and 4.5 g L^–1^ glucose (Corning) supplemented with 10% fetal bovine serum (Avantor) and a 100× combination solution of 10,000 IU mL^–1^ penicillin and 10,000 μg mL^–1^ streptomycin (Corning) at 37 °C, 5% CO_2_. Mammalian cultures at 70–80% confluency were used for cell viability and cytotoxicity experiments.

### Growth Inhibition

Growth inhibition assays were performed by microbroth dilution following the guidelines set by the CLSI.^58^ Briefly, *Candida* spp. was maintained on BBL Sabouraud dextrose agar (SDA) at 35 °C for 48 h. Inoculums were standardized to 2 × 10^3^ CFU mL^–1^ in RPMI-1640 medium. Wells were treated with penta-*O*-galloyl-β-d-glucose (PGG, 0.0039–8.0 μg mL^–1^) (≥96% purity, Sigma Aldrich), positive controls (0.0039–8.0 μg mL^–1^), or an equivalent volume of the DMSO vehicle. Percent growth inhibition was determined by comparing treatments to the DMSO vehicle control. MIC was determined by measuring OD_600_ with a BioTek Cytation3 plate reader after incubation (35 °C) with treatment for 48 h. MIC was defined as the lowest treatment concentration with >90% growth inhibition. The IC_50_ was determined by the lowest treatment concentration with >50% growth inhibition. Positive controls were fluconazole (MP Biomedicals), ketoconazole, and amphotericin B (Alfa Aesar). The negative control was untreated culture. A media blank was used to monitor for media contamination. Tests were repeated in duplicate with three technical replicates per treatment and on different days.

For media supplementation experiments, magnesium sulfate (Alfa Aesar), manganese sulfate, zinc sulfate, and calcium chloride (Ward Science) were dissolved in RPMI-1640 media and, after 24 h, added at a final concentration of 1 mM. Growth inhibition was measured 24 h after media supplementation. Percent growth inhibition was determined by comparing treatments to vehicle controls. Media supplementation experiments were repeated in triplicate with four technical replicates per treatment and on different days.

For iron supplementation assays, growth inhibition assays were performed by microbroth dilution following the guidelines set by the CLSI.^58^ A solution of iron (II) sulfate and iron (III) sulfate (VWR) was dissolved in deionized water to a concentration of 10 mM and added to treated wells at the beginning of incubation. Growth inhibition was measured after 48 h. Percent growth inhibition was determined by comparing treatments to vehicle controls. Iron supplementation experiments were repeated in duplicate with three technical replicates per treatment and on different days.

### Method for Concentration-Response Matrixes (Checkerboard Assays)

Cells were grown overnight in yeast extract peptone dextrose (YPD) at 30 °C under shaking conditions (200 rpm). Checkerboard assays were set up in 96-well flat-bottom microtiter plates (Sarstedt) in a total well volume of 0.1 mL. Cell inoculums were prepared from saturated overnight cultures at 2 × 10^4^ CFU mL^–1^, which is 10-fold higher than the inoculums used in the CLSI MICs to increase the dynamic range of the assay. Twofold serial dilutions of compounds in RPMI–1640 medium were prepared along the *y-* and *x-axes*. Plates were incubated at 30 °C in static conditions, and growth was assessed by measuring absorbance (OD_600_) after 48 h. Growth was corrected for the medium background and normalized to the no-drug controls. All assays were performed in technical duplicates in two biological replicates. Data was quantitatively displayed using Java Treeview3. The fractional inhibitory concentration index (FICI) at 90% growth inhibition was calculated to evaluate the interaction of the two drugs in combination, with values <0.5 indicating a synergistic interaction.^59^

### Method for the Ergosterol Binding Assay

Ergosterol binding was measured using a previously described method,^60^ with some modifications. Briefly, ergosterol (TCI) was pulverized with a sterile pestle and mortar and then dissolved in 100% ethanol. The resultant emulsion was warmed at 37 °C for 15 min and then sonicated for 45 min prior to use. PGG (Sigma Aldrich) and the positive control amphotericin B (Alfa Aesar) were serially diluted to a final concentration equal to 1× – 4× the MIC of PGG (1.0–4.0 μg mL^–1^). Growth inhibition was determined in the presence and absence of 80 μg mL^–1^ of exogenous ergosterol by broth microdilution following the guidelines set by the CLSI.^58^ Percent growth inhibition was measured after 24 h of incubation by comparing treatments to vehicle controls. The negative control was untreated culture. A media blank was used to monitor for contamination. Experiments were repeated in triplicate with three technical replicates per treatment and on different days.

### Concentration-Response Assays of *C. albicans* Efflux and Iron Homeostasis Mutants

Strains were grown overnight in YPD at 30 °C under shaking conditions (200 rpm). Cell inoculums were prepared in RPMI-1640 medium at 2 × 10^4^ CFU mL^–1^, which is 10-fold higher than the inoculums used in the CLSI MICs to increase the dynamic range of the assay. Concentration-response assays were performed in RPMI-1640 medium using a broth microdilution protocol, as previously described.^61^ Plates were incubated at 30 °C in static conditions for 48 or 72 h, as indicated. For the efflux mutants, growth was measured by absorbance (OD_600_). For the iron homeostasis mutants, growth was measured by Alamar blue (Invitrogen) fluorescence at 530/590 nm (excitation/emission) to circumvent issues with optical density quantification of filamentous cells. Growth was corrected for the medium background and normalized to the no-drug wild-type controls. All assays were performed in technical duplicates in two biological replicates. Data was quantitatively displayed using Java Treeview3.

### 2,2′-Bipyridyl Test

The 2,2′-bipyridyl test was followed and analyzed as outlined previously^27^ and modified for a 96-well plate format. Briefly, 2,2′-bipyridyl (Sigma Aldrich) interacts with free iron (II) to produce a red-colored complex [Fe(bipy)_3_]^2+^ with absorbance at 520 nm. A 0:1 solution of iron (III):ligand was prepared at starting concentrations of 10 μM, adjusted to 2.0 pH, and allowed to equilibrate at room temperature for 1 h. This was repeated for 1:1, 2:1, 3:1, etc. iron (III):ligand mixtures. A solution of 2,2′-bipyridyl was added to the iron (III):ligand mixture and mixed at 650 rpm for 5 min. Absorbance was read with a Cytation3 plate reader with path length correction selected. Solutions of ligand only were used as a blank. The positive control was epigallocatechin-gallate (EGCG, Cayman Chemical). The negative control was a solution of only 2,2′-bipyridyl. The molar absorptivity of [Fe(bipy)_3_]^2+^ was previously found to be 8200 M^–1^ cm^–1^.^27^ The number of Fe^2+^ ions produced and the number of electron transfers that occurred were then calculated with the following equation:
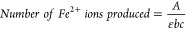
where *A* = absorbance, ε = molar absorptivity of [Fe(bipy)_3_]^2+^ (8200 M^–1^ cm^–1^), *b* = path length of the beam (1 cm), and *c* = concentration of the ligand (0.00001 M).

### Method for Job’s Method

In a 384-well plate, the ligand and metal were added to a 1:1 DMSO:H_2_O solution in varying mole ratios to a total ligand and metal concentration of 0.76 mM (PGG) from 7.6 mM stock concentrations to a total volume of 100 μL. The mixtures were incubated at room temperature for approximately 5 min, and each well was scanned between 400 and 750 nm at 2 nm increments.

### Cell Viability and Cytotoxicity

#### Chemical-Induced Cytotoxicity

The mammalian culture was standardized to 4 × 10^3^ cells per 100 μL. Cells were allowed to attach and incubate for 24 h before growth media was replaced with treatment media (PGG, 16–512 μg mL^–1^) or an equivalent volume of the DMSO vehicle, followed by incubation for 24 h. Plates were then evaluated with an LDH cytotoxicity assay (G Biosciences) and processed according to the manufacturer’s protocol for chemical-induced cytotoxicity. Experiments were repeated in triplicate with three technical replicates per treatment and on different days.

### Cell Viability/Proliferation

ATP content as a marker for the relative viable cell number was measured with the CellTiter-Glo Luminescent Cell Viability assay (Promega Corporation). The mammalian culture was standardized at 4 × 10^3^ cells per 100 μL in a Costar 3610 white plate, clear bottom 96-well plates (Corning). Cells were allowed to attach and incubate for 24 h before growth media was replaced with treatment media (PGG, 16–512 μg mL^–1^) or an equivalent volume of DMSO, followed by incubation for 24 h. After incubation with treatment, plates were equilibrated to room temperature for 1 h, and then 100 μL of CellTiter-Glo reagent was added to each well and mixed for 2 min. Plates were allowed to incubate at room temperature for 10 min before luminosity was measured with an integration time of 1 s via a BioTek Cytation3 plate reader. The relative viable cell number was measured by comparing treatment samples to untreated controls. Experiments were repeated in triplicate with three technical replicates per treatment and on different days.

### Statistical Analysis

Where applicable, data were analyzed by two-way ANOVA with Tukey’s multiple comparison test using GraphPad Prism version 9.30 for Windows, GraphPad Software, San Diego, California, USA, www.graphpad.com. A *p*-value <0.05 was considered significant.

## Data Availability

ChemDraw Professional version 16.0.1.4 (77) was used for chemical structures.

## Supporting Information Available

Experimental methods for concentration-response matrixes; effects of penta-*O*-galloyl-β-d-glucose upon human cytotoxicity and cell viability; media supplementation assays; Job’s method spectra; expanded MIC graphs with additional positive controls; and table with the additional *Candida albicans* strains and genotype (PDF)
