# Supplementary material for: Potent Antifungal Activity of Penta-O-galloyl-β-d-Glucose against Drug-Resistant Candida albicans, Candida auris, and Other Non-albicans Candida Species
Source: ACS Infect Dis. 2023 Aug 22;9(9):1685–94. doi: 10.1021/acsinfecdis.3c00113 (PMC10496123; doi:10.1021/acsinfecdis.3c00113)
Supplement: Supplementary file 1 — id3c00113_si_001.pdf [file id3c00113_si_001.pdf]

Supplementary Information

Potent antifungal activity of penta-*O*-galloyl-β-D-glucose against drug-resistant *Candida albicans*, *Candida auris*, and other non-*albicans Candida* species

Lewis Marquez[a,b], Yunjin Lee[c], Dustin Duncan[c,d,†], Luke Whitesell[c], Leah E. Cowen[c], Cassandra Quave[e,f,*]

[a]Molecular and Systems Pharmacology, Laney Graduate School, Emory University, Atlanta, Georgia, USA 30322

[b]Jones Center at Ichauway, Newton, Georgia, USA 39870

[c]Department of Molecular Genetics, University of Toronto, Toronto, Ontario M5G 1M1, Canada

[d]Department of Chemistry, Brock University, St. Catharines, Ontario, Canada, L2S 3A1

[e]Center for the Study of Human Health, Emory University, Atlanta, Georgia, USA 30322

[f]Department of Dermatology, Emory University, Atlanta, Georgia, USA 30322

[†]Current address: Department of Chemistry, Brock University

Running title: Potent antifungal activity of pentagalloyl glucose

*Corresponding author: cquave@emory.edu

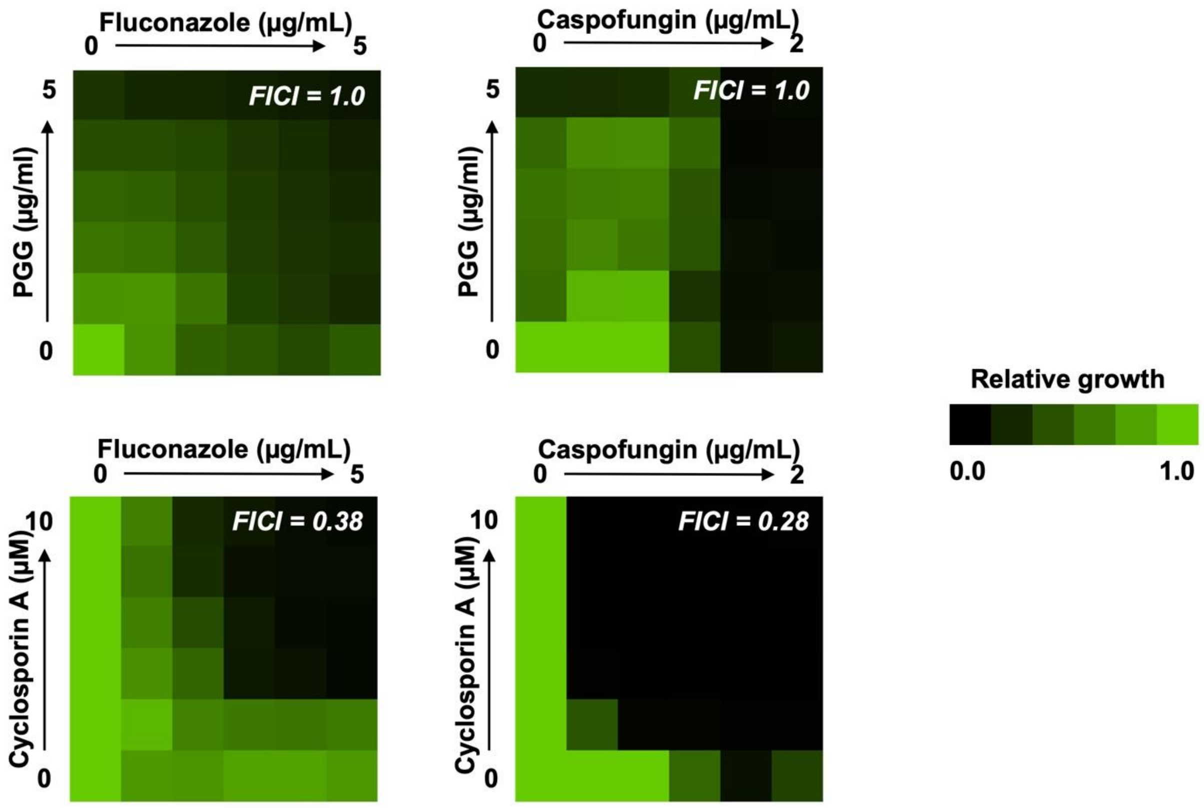

Supplemental Figure S1. Penta-*O*-galloyl-β-D-glucose does not synergize with conventional antifungals. Wild-type *C. albicans* (ATCC 90028) and the azole-tolerant clinical isolate (CaCi-2) were grown overnight in YPD and subject to dose-response matrixes with caspofungin or fluconazole, respectively, in combination with penta-*O*-galloyl-β-D-glucose (PGG). Positive control was cyclosporin A. Growth (OD_{600}) was measured after 48 hours of incubation at 30°C, averaged between technical duplicates, and normalized to the drug-free control (see colour bar). All assays were performed in biological duplicate. The fractional inhibitory concentration index (FICI) at 90% growth inhibition was calculated to evaluate the interaction of the two drugs in combination, with values <0.5 indicating a synergistic interaction.

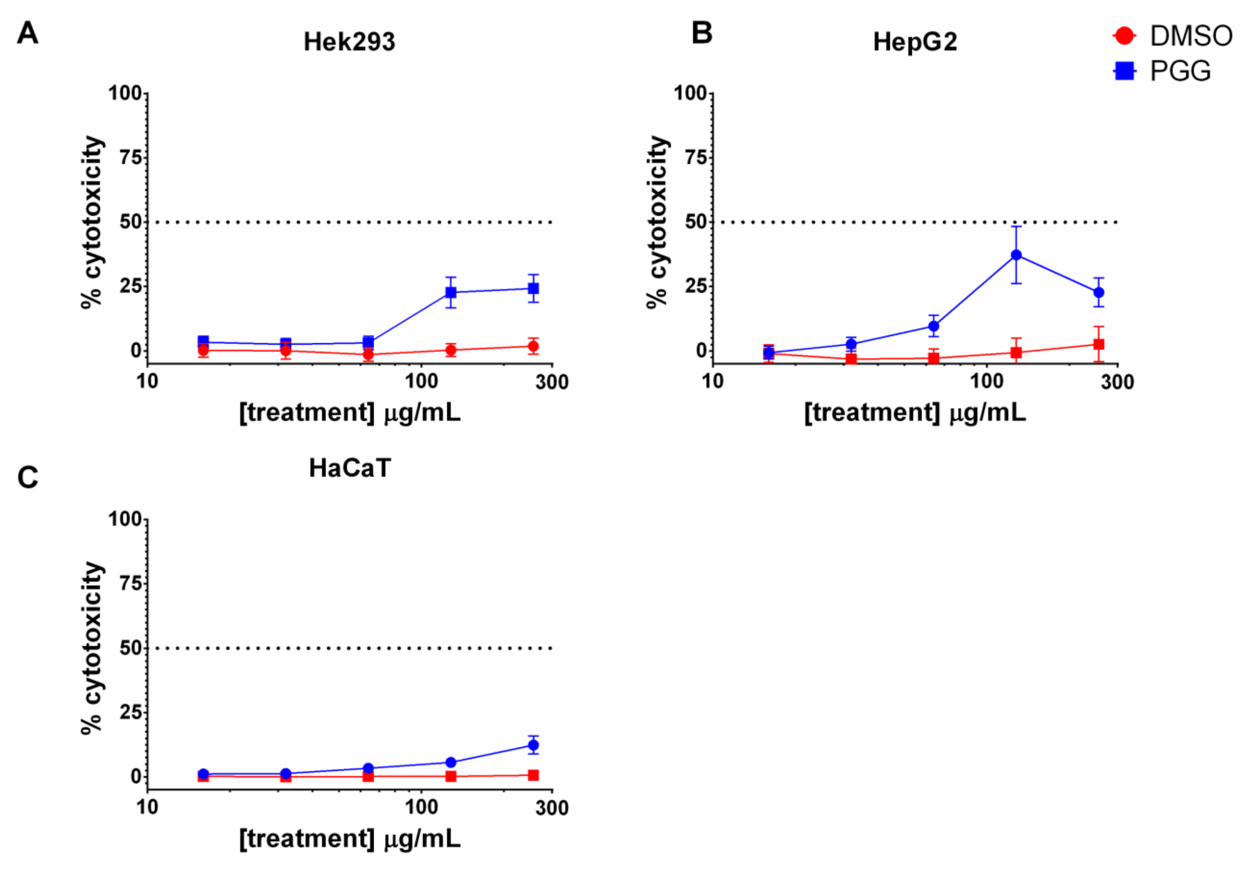

Supplemental Figure S2. Penta-*O*-galloyl-β-D-glucose displays low *in vitro* cytotoxicity.

Cytotoxicity of PGG against **(A)** Hek293, **(B)** HepG2, and **(C)** HaCaT cells. Human cells were incubated with PGG (16 – 256 µg mL^{-1}) for 24 hours and measured for cell lysis. The data are plotted as mean % cytotoxicity ± SD as compared to total lysis controls from three independent experiments. Dotted line represents 50% cytotoxic concentration.

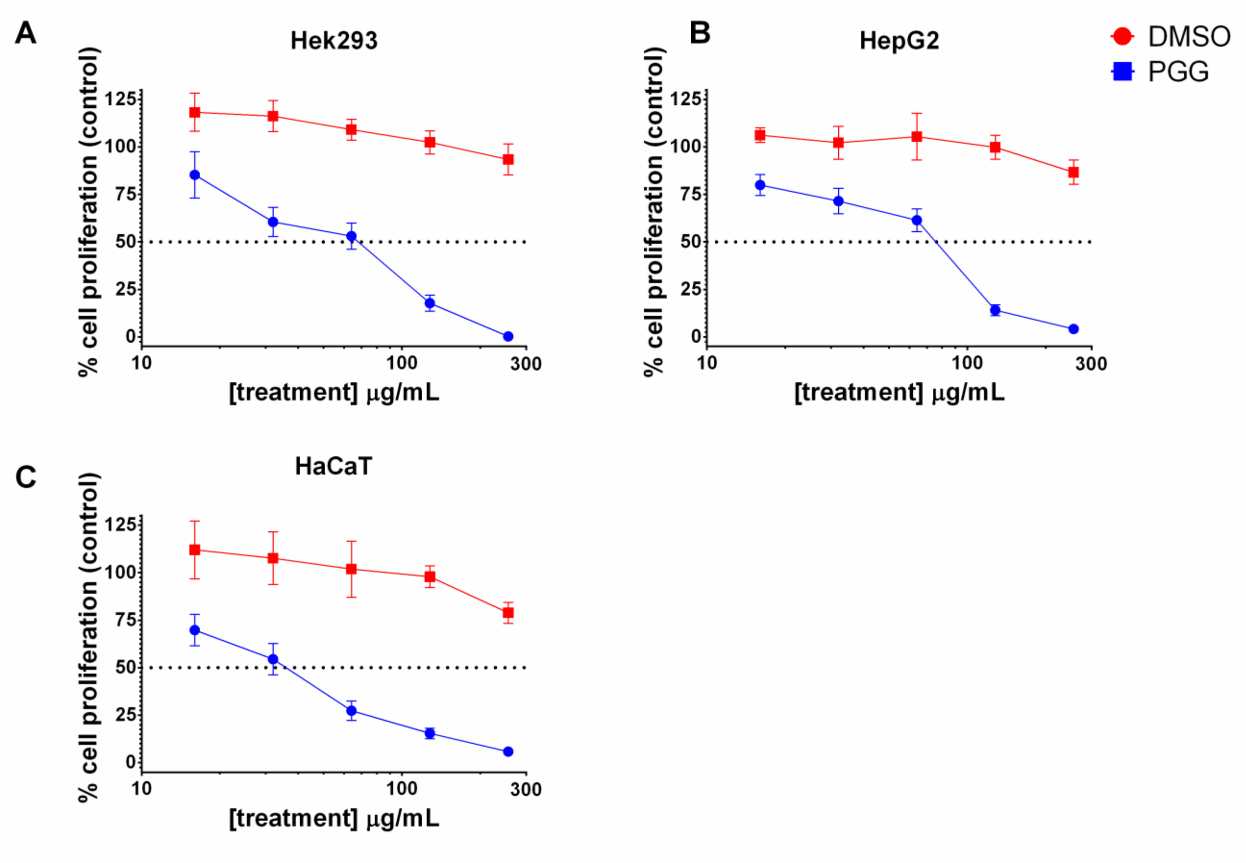

Supplemental Figure S3. Penta-*O*-galloyl-β-D-glucose reduces cell proliferation. Cell proliferation of PGG against **(A)** Hek293, **(B)** HepG2, and **(C)** HaCaT cells. Human cells were incubated with PGG (16 – 256 µg mL^{-1}) for 24 hours and measured for ATP production as a marker for proliferation. The data are plotted as mean % cell proliferation ± SD as compared to untreated controls from three independent experiments. Dotted line represents 50% inhibitory concentration.

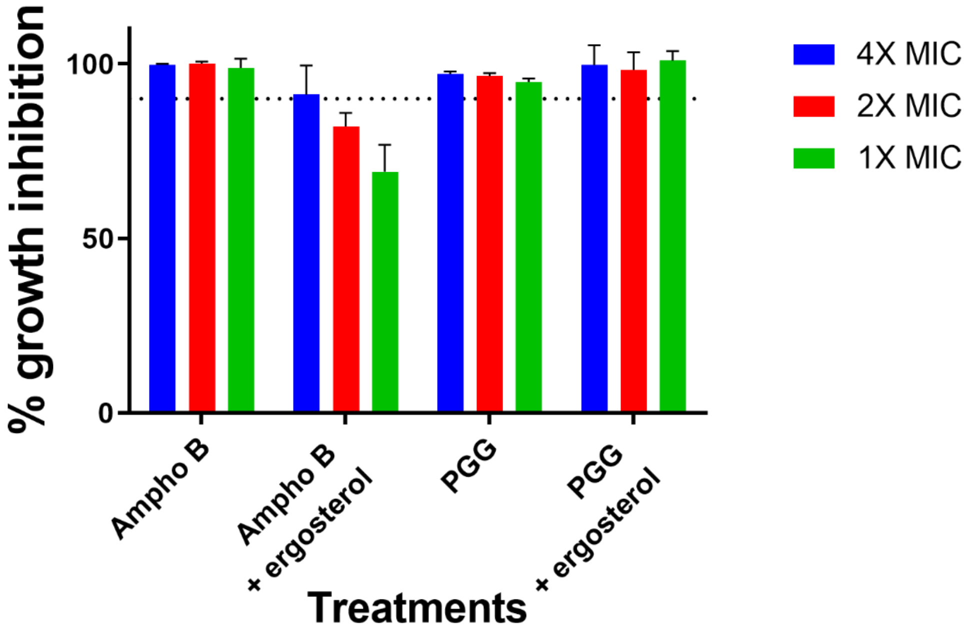

Figure S4. Growth inhibition of penta-*O*-galloyl-β-D-glucose against *C. albicans* not affected by additional ergosterol. *C. albicans* was treated with PGG or amphotericin B, known to bind to fungal ergosterol, at varying MIC concentrations (1×, 2×, and 4×) for 24 hours, in the absence or presence of exogenous ergosterol (80 μg mL^{-1}). After 24 hours, treatment groups were measured for growth inhibition. The data are plotted as mean % growth inhibition ± SD as compared to vehicle controls from three independent experiments. Dotted line represents 90% growth inhibition.

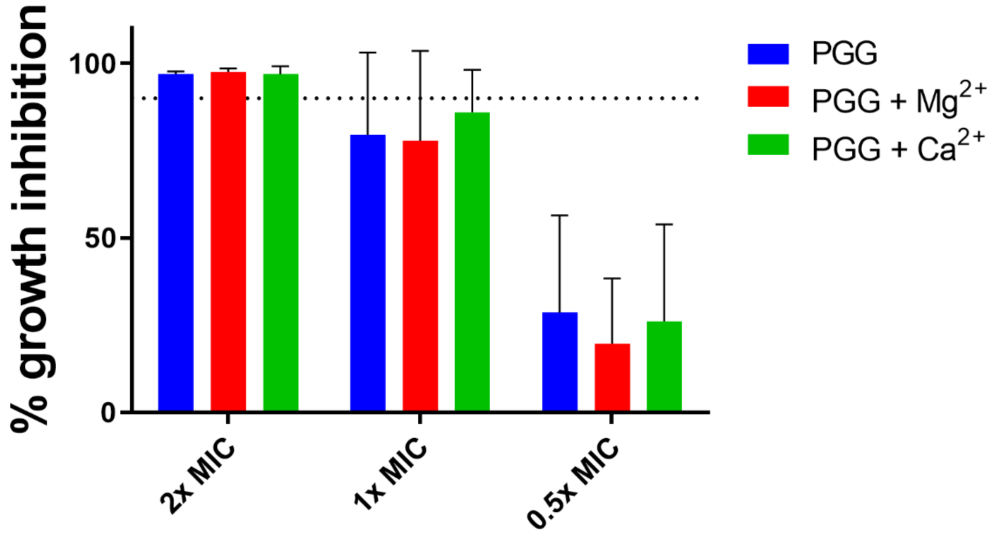

Figure S5. Effect of supplemental cations on growth inhibition of penta-*O*-galloyl-β-D-glucose against *C. albicans*. *C. albicans*.90028 was treated with PGG alone and in combination with 1 mM magnesium (Mg^{2+}) or 1 mM calcium (Ca^{2+}). The data are plotted as mean % growth inhibition ± SD as compared to vehicle controls from two independent experiments. Dotted line represents 90% growth inhibition.

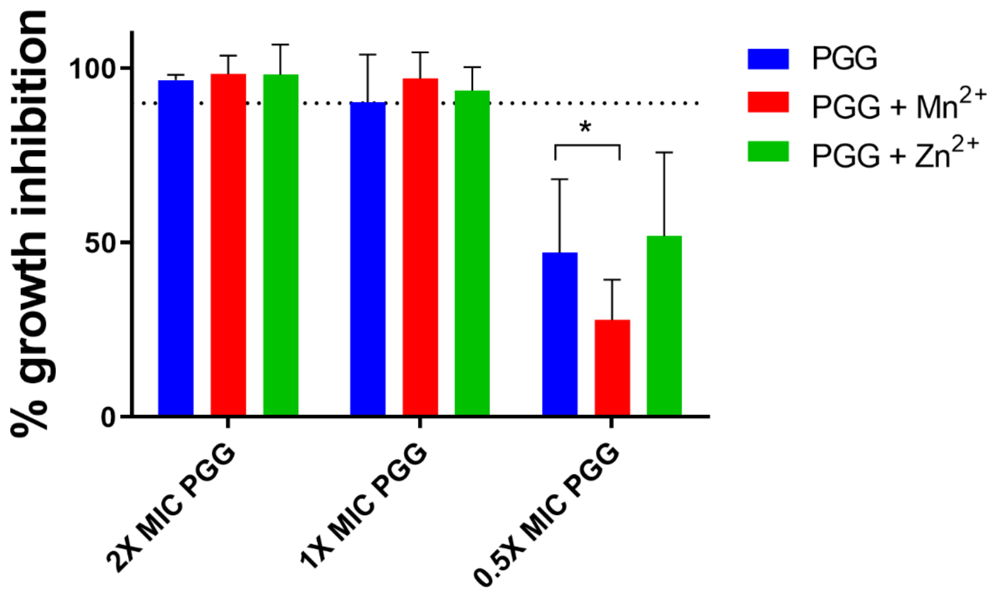

Figure S6. Effect of supplemental metal ions on growth inhibition of penta-*O*-galloyl-β-D-glucose against *C. albicans*. *C. albicans* 90028 was treated with PGG alone and in combination with 1 mM manganese (Mn^{2+}) or 1 mM zinc (Zn^{2+}). The data are plotted as mean % growth inhibition ± SD as compared to vehicle controls from two independent experiments. Dotted line represents 90% growth inhibition. Statistical analysis was done using 2-way ANOVA with multiple comparisons. The symbol (*) signifies a p-value ≤ 0.05.

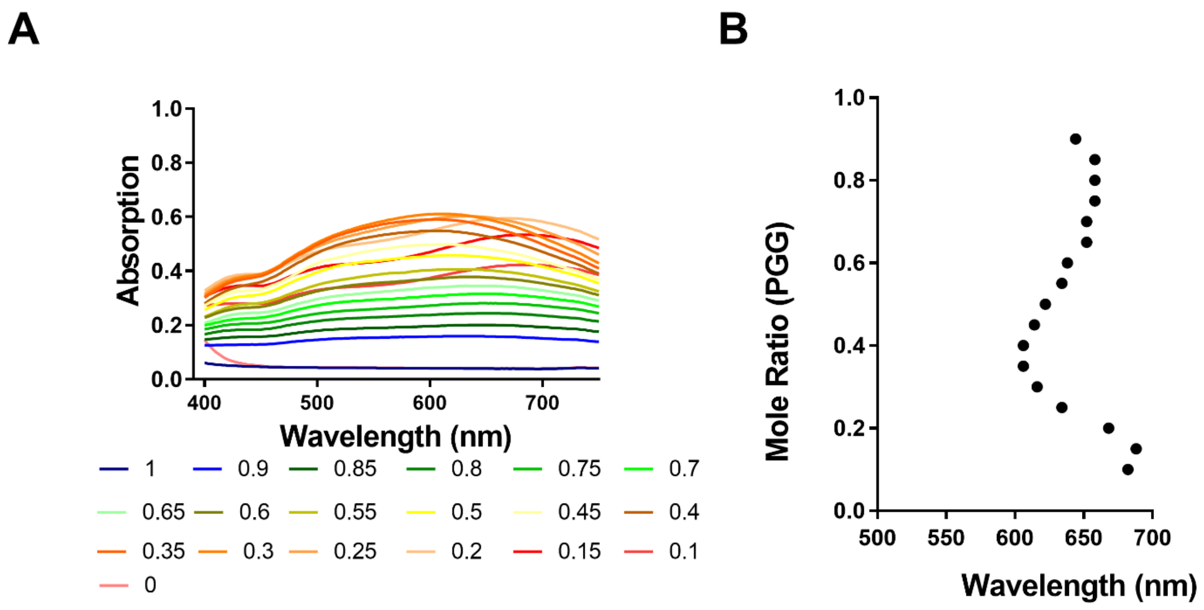

Figure S7. Penta-*O*-galloyl-β-D-glucose forms multiple complexes with various maximum absorptions. (A) Wavelength scan of PGG at mole fractions varying from 0 – 1 with $FeCl_3$. [PGG]+[Fe^{3+}] = 0.76 mM. Solutions of ligand and metal were incubated for approximately 5 minutes before measuring the UV/Vis spectrum between 400 nm and 750 nm for each well at 2 nm steps. **(B)** Maximum wavelengths for the wavelength scan at different mole ratios of PGG and $FeCl_3$.

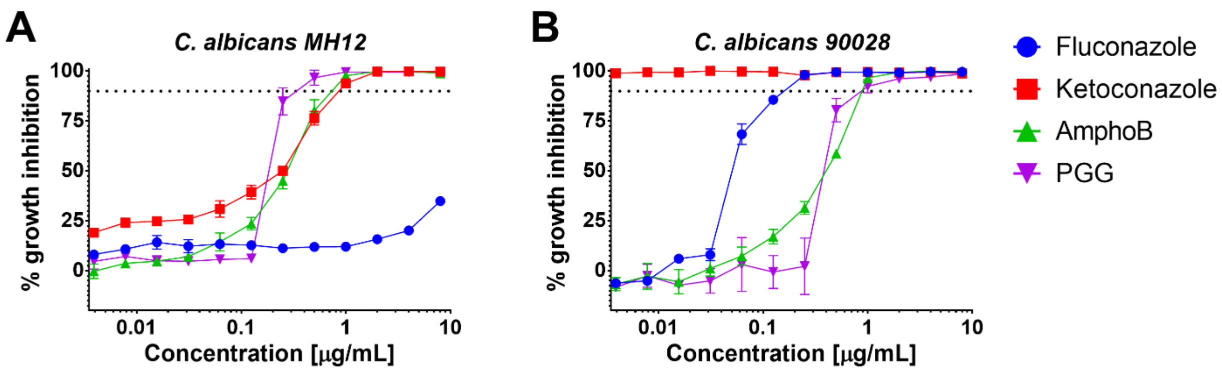

Figure S8. Penta-*O*-galloyl-β-D-glucose displays activity against two *C. albicans* isolates. Growth inhibition of PGG against **(A)** MH12 **(B)** ATCC90028. Fluconazole, ketoconazole, and amphotericin B were used as positive controls. The data are plotted as mean % growth inhibition ± SD as compared to DMSO vehicle from two independent experiments. Dotted line represents 90% growth inhibition.

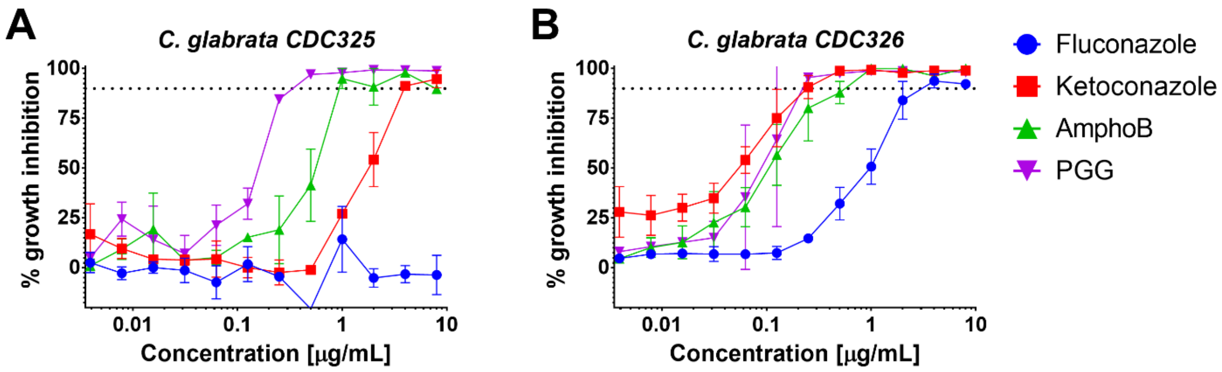

Figure S9. Penta-*O*-galloyl-β-D-glucose displays activity against two *C. glabrata* isolates. Growth inhibition of PGG against **(A)** CDC325 **(B)** CDC326. Fluconazole, ketoconazole, and amphotericin B were used as positive controls. The data are plotted as mean % growth inhibition ± SD as compared to DMSO vehicle from two independent experiments. Dotted line represents 90% growth inhibition.

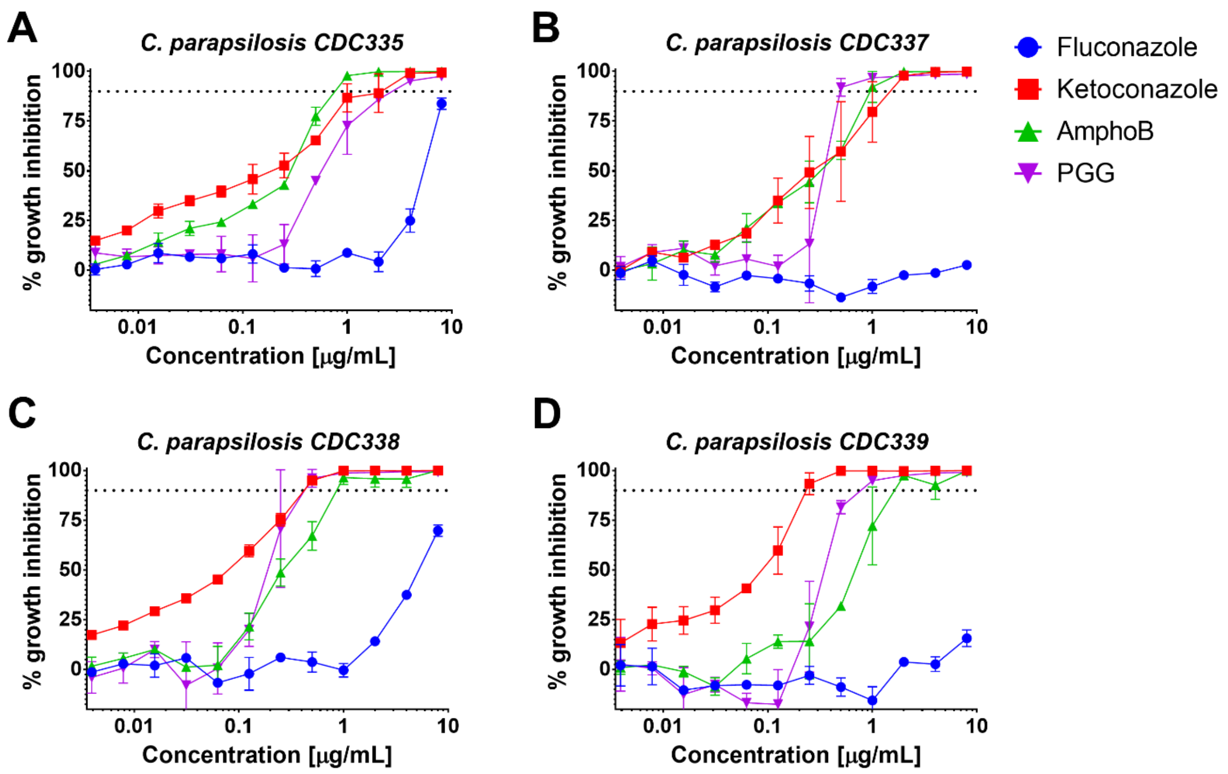

Figure S10. Penta-*O*-galloyl-β-D-glucose displays activity against *C. parapsilosis* panel. Growth inhibition of PGG against **(A)** CDC335, **(B)** CDC337, **(C)** CDC338, and **(D)** CDC339. Fluconazole, ketoconazole, and amphotericin B were used as positive controls. The data are plotted as mean % growth inhibition ± SD as compared to DMSO vehicle from two independent experiments. Dotted line represents 90% growth inhibition.

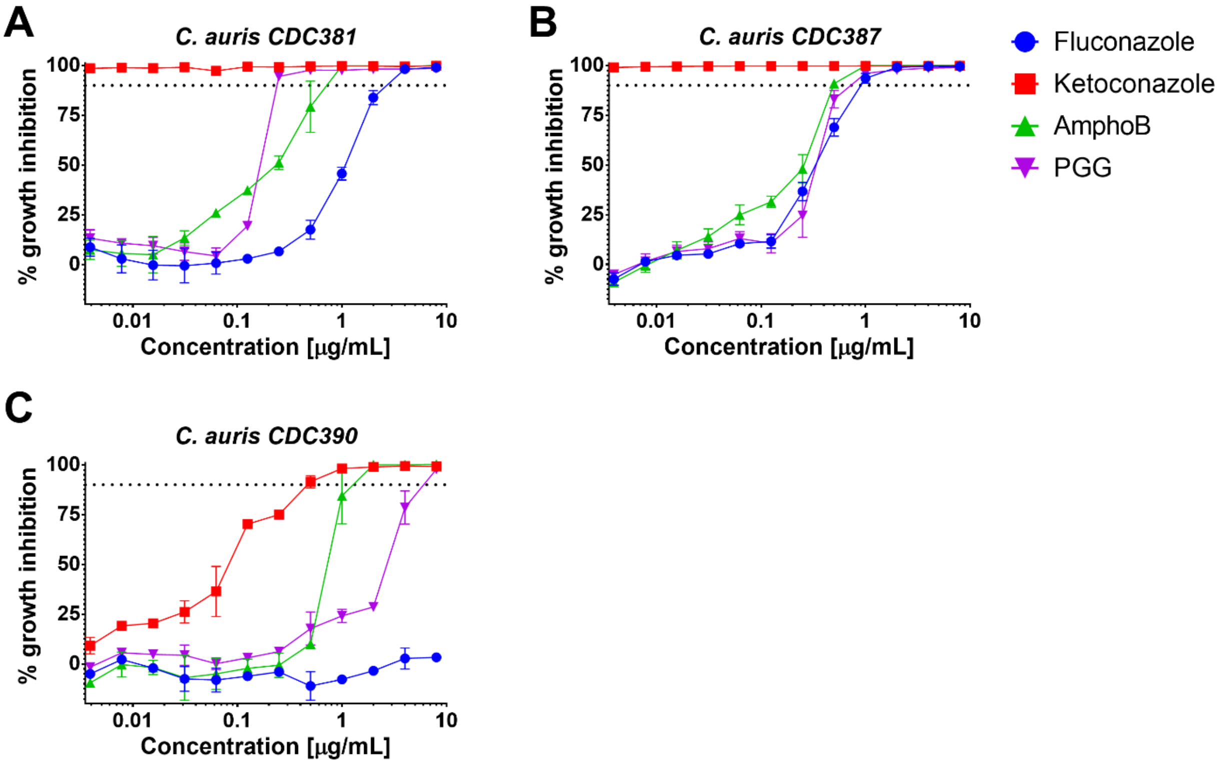

Figure S11. Penta-*O*-galloyl-β-D-glucose displays activity against *C. auris* panel. Growth inhibition of PGG against **(A)** CDC381, **(B)** CDC387, and **(C)** CDC390. Fluconazole, ketoconazole, and amphotericin B were used as positive controls. The data are plotted as mean % growth inhibition ± SD as compared to DMSO vehicle from two independent experiments. Dotted line represents 90% growth inhibition.

Table S1. Additional *Candida albicans* strains and genotype.

**Strain Name**	**Genotype**	**Source**
C. albicans SN95	*arg/arg4 his1/his1 URA3/ura3::imm*^{434} *IRO1/iro1::imm*^{434}	1
C. albicans SN152	*arg4 /arg4 , leu2 /leu2 ::LEU2(Ca), his1 /his1 ::HIS1(Ca), URA3/ura3 , IRO1/iro1*	1
C. albicans CAF2-1	*ura3::imm*^{434}*/URA3*	2
C. albicans DSY1024 *cdr1Δ/cdr1Δ cdr2Δ/cdr2Δ mdr1Δ/mdr1Δ flu1Δ/ flu1Δ*	*cdr1 hisG/cdr1 ::hisG cdr2 ::hisG/cdr2 ::hisG camdr1 ::hisG/camdr1 ::hisG flu1 ::hisG/flu1 ::hisG-URA3- hisG*	3
C. albicans ire1Δ/Δ	*arg4/arg4 his1/his1 URA3/ura3::imm*^{434} *IRO1/iro1::imm*^{434} *ire1Δ::HYGB/ ire1Δ::HYGB*	4
C. albicans hap43Δ/Δ	*arg4 /arg4 , leu2 /leu2 ::LEU2(Ca), his1 /his1 ::HIS1(Ca), URA3/ura3 , IRO1/iro1 , hap43 / hap43*	5
C. albicans rim101Δ/Δ	*arg4 /arg4 , leu2 /leu2 ::LEU2(Ca), his1 /his1 ::HIS1(Ca), URA3/ura3 , IRO1/iro1 , rim101 / rim101*	5

References

(1) Noble, S. M.; Johnson, A. D. Strains and strategies for large-scale gene deletion studies of the diploid human fungal pathogen Candida albicans. *Eukaryot Cell* **2005**, *4* (2), 298-309. DOI: 10.1128/ec.4.2.298-309.2005 From NLM.

(2) Fonzi, W. A.; Irwin, M. Y. Isogenic strain construction and gene mapping in Candida albicans. *Genetics* **1993**, *134* (3), 717-728. DOI: 10.1093/genetics/134.3.717 From NLM.

(3) Calabrese, D.; Bille, J.; Sanglard, D. A novel multidrug efflux transporter gene of the major facilitator superfamily from Candida albicans (FLU1) conferring resistance to fluconazole. *Microbiology* **2000**, *146* (11), 2743-2754. DOI: https://doi.org/10.1099/00221287-146-11-2743.

(4) Lee, Y.; Hossain, S.; MacAlpine, J.; Robbins, N.; Cowen, L. E. Functional genomic analysis of Candida albicans protein kinases reveals modulators of morphogenesis in diverse environments. *iScience* **2023**, *26* (3), 106145. DOI: 10.1016/j.isci.2023.106145 From NLM.

(5) Homann, O. R.; Dea, J.; Noble, S. M.; Johnson, A. D. A Phenotypic Profile of the Candida albicans Regulatory Network. *PLoS Genet.* **2009**, *5* (12), e1000783. DOI: 10.1371/journal.pgen.1000783.
